# Black soybeans protect human keratinocytes from oxidative stress‐induced cell death

**DOI:** 10.1002/fsn3.842

**Published:** 2018-10-20

**Authors:** Young Yoon, Yoon‐Mi Lee, Sooji Song, Yu Young Lee, Kyung‐Jin Yeum

**Affiliations:** ^1^ Division of Food Bioscience College of Biomedical and Health Sciences Konkuk University Chungju‐si Korea; ^2^ Nanotechnology Research Center Konkuk University Chungju‐si Korea; ^3^ Department of Central Area National Institute of Crop Science Rural Development Administration Suwon Korea; ^4^ Institute of Biomedical and Health science Konkuk University Chungju‐si Korea

**Keywords:** anthocyanin, apoptosis, caspases, HO‐1, Nrf2

## Abstract

Black soybeans are functional foods containing a variety of bioactives such as isoflavones, carotenoids, tocopherols, phenolic acid as well as anthocyanins. Here, we examined whether Cheongja#3 black soybean extract has a protective effect on oxidative stress‐induced cell death in human keratinocytes HaCaT. First, we identified fat‐soluble bioactives in three varieties of soybean extracts (Saedanbaek, Daechan, and Cheongja#3). In particular, black soybean Cheongja#3 had high amounts of lutein than other varieties. We demonstrated that Cheongja#3 extract reduced intracellular reactive oxygen species levels in HaCaT cells. Furthermore, Cheongja#3 protected cells from hydrogen peroxide (H_2_O_2_)‐induced oxidative stress and triggered cell death determined by cell viabilities and apoptotic caspase activities. Next, we identified the underlying mechanism is due to increased Nrf2 antioxidant system by Cheongja#3, thus increasing the expression of heme oxygenases (HO)‐1. These results indicated that Cheongja#3 soybean extract has protective role against oxidative stress by upregulating the Nrf‐2 antioxidant system in human keratinocyte HaCaT cells.

## 1 INTRODUCTION

Constant generation of reactive oxygen species (ROS) at low levels is essential for cells for avoiding extracellular invaders and maintaining cellular signaling. However, oxidative stress caused by over production of ROS induces cell damage of macromolecules such as DNA, proteins, and lipids (Reuter, Gupta, Chaturvedi, & Aggarwal, 2010). Accumulation of such cell damage leads to chronic diseases including cancer, cardiovascular diseases, neurodegenerative diseases, and others (Ray, Huang, & Tsuji, 2012). Various literatures have emphasized importance of antioxidants on attenuating oxidative stress and its associated chronic diseases (Baynes, 1991; Osawa & Kato, 2005).

Skin epidermis serves as barrier of body from environmental toxins and oxidative stress. Particularly, keratinocytes are predominant cells of the epidermis. Responding to hydrogen peroxide (H_2_O_2_) which plays a pivotal role among ROS, keratinocytes trigger apoptosis determined by released cytochrome c, cleaved caspases activities, and proapoptotic gene expressions (Zuliani et al., 2005). The oxidative damaged keratinocytes contribute the pathogenesis of skin‐related diseases such as psoriasis, skin aging, and skin cancer (Bae et al., 2014; Kohen, 1999; Liu et al., 2011), thus emerging studies that antioxidants protect against H_2_O_2_‐induced apoptosis in human keratinocytes HaCaT (Bae et al., 2014; Nguyen, Kim, & Lee, 2013; Seo & Jeong, 2015).

Nuclear transcription factor erythroid‐2‐like factor 2 (Nrf2) is one of the major antioxidant systems that protect cells from oxidative stress (Motohashi & Yamamoto, 2004). Under normal condition, Nrf2 action is blocked by Keap1. Responding to extracellular stimuli such as UV light, oxidative stress, and hypoxia, Nrf2 is released from Keap1, thus translocating to the nucleus to bind to its downstream genes containing antioxidant response element (ARE) consensus sequence (Seo & Jeong, 2015). Heme oxygenase‐1 (HO‐1) is an antioxidant enzyme which contains an ARE site, and functions in the degradation of heme to bilirubin, carbon monoxide, and iron (Seo et al., 2011). Previous studies have reported that natural products from food sources induce antioxidant activity through upregulation of Nrf2‐mediated HO‐1 expression (Hseu et al., 2012; Nguyen et al., 2013; Seo et al., 2011).

Soybeans are rich in proteins, carbohydrates, dietary fiber, and phytochemicals. Isoflavones are unique components in soybeans and offer a variety of health benefits against obesity, cancer, diabetes, kidney diseases, osteoporosis, and cardiovascular disease (Anderson & Major, 2002; Anderson, Smith, & Washnock, 1999). In particular, black soybeans have been reported to contain even more nutrients including anthocyanins in their seed coat (Liao, Chen, & Yang, 2005). Their biological activities including antioxidative and anti‐inflammatory effects help to reduce the risk of cancer and metabolic disorders (Ganesan & Xu, 2017). Cheongja#3 is a cultivar of black soybean which is well known to contain high amounts of anthocyanins as well as tocopherols (Lee et al., 2009; Lee, Park, et al., 2015). Several previous studies have shown that Cheongja#3 had antiobesity effects in cells, mice, and humans (Jeon, Lee, & Cheon, 2015; Kim, Kim, et al., 2012; Kim et al., 2015; Lee, Sorn, Park, & Park, 2016), as well as neuroprotective effects (Bhuiyan, Kim, Ha, Kim, & Cho, 2012; Kim, Chung, et al., 2012). However, there has been a lack of information on the protective effects of Cheongja#3 with respect to oxidative damage in human keratinocyte HaCaT. Here, we tested the effect of Cheongja#3 on reducing oxidative stress‐induced cell death and examined underlying mechanism of such action in HaCaT cells.

## 2 METHODS AND MATERIALS

### 2.1 Preparation of soybean extract

Three soybean cultivars (Saedanbaek, Daechan, Cheongja#3) were newly developed by National Institute of Crop Science as previously reported (Lee, Choi, et al., 2015; Lee et al., 2009; Min et al., 2015), and all soybeans were provided form the National Institute of Crop Science. These soybeans were grounded into powder at 500 g for 5 min, respectively. Forty grams of each powdered soybean was extracted in 500 ml of 40% ethanol solution (EtOH) for 24 hr. After repeating three times, the solutions were filtered and freeze dried.

### 2.2 Reagents

Dulbecco's modified Eagle's medium (DMEM), antibiotic antimycotic solution, hydrogen peroxide (H_2_O_2_), 3‐(4,5‐Dimethylthiazol‐2‐yl)‐2,5‐Diphenyltetrazolium Bromide (MTT), dimethyl sulfoxide (DMSO), and 2,2‐Diphenyl‐1‐picrylhydrazyl (DPPH) were purchased from Sigma‐aldrich (St. louis, MO, USA). Fetal bovine serum (FBS) was obtained from Gibco (Waltham, MA, USA). RIPA buffer was purchased from Thermo Fisher (Waltham, MA, USA). Phosphate inhibitor and protease inhibitor were purchased from Gen DEPOT (Barker, TX, USA). DCF‐DA kit was purchased from Abcam (Cambridge, UK), and caspases‐3 was from Cayman chemicals (Ann Arbor, MI, USA). Antibodies against caspase‐3, caspase‐6, and caspase‐7 were obtained from Cell signaling Technology (Danvers, MA, USA). Antibody against Nrf2 was from Thermo Fisher scientific. Antibodies against heme oxygenase (HO), Lamin B, and β‐actin were from Santa Cruz (Dallas, TX, USA). ECL prime was purchased from GE Healthcare life sciences (Buchinghamshire, UK).

### 2.3 Cell culture

HaCaT cells were kindly provided from Dr. Ji‐Hong Lim (Department of Integrated Biosciences, Konkuk University) and cultured in DMEM containing 10% FBS and antibiotics (100 units/ml penicillin, 100 μg/ml streptomycin, and 250 ng/ml amphotericin B) and incubated at 5% CO_2_ and 37°C in a humidified air.

### 2.4 Ultra performance liquid chromatographic (UPLC) analysis

Fat‐soluble micronutrients were extracted by using the slightly modified Folch method (Folch, Lees, & Sloane Stanley, 1957) and analyzed by previously reported UPLC method (Delpino‐Rius et al., 2014). The UPLC (ACQUITY UPLC I‐Class, Waters Co., Milford, MA, USA) system was equipped with a BEH C18 column (1.7 μm, 2.1 × 50 mm, Waters Co.), binary pump delivery system, autosampler, and photodiode array detector. The mobile phase A was acetonitrile/methanol (7:3, v/v), and the mobile phase B was water. Each sample was injected into the BEH C18 column (1.7 μm, 2.1 × 50 mm). The gradient conditions are described in Table 1. γ‐Tocopherol (at 292 nm) and lutein (at 450 nm) were quantified by each standard curve. Each peak was confirmed by retention time and its unique spectrum. The interassay coefficient of variation (CV) was under 4% (*n* = 10), and the intraassay CV was under 4% as well (*n* = 10).

**Table 1:** Chromatographic conditions of UPLC gradient elution methods. A gradient elution was carried out with acetonitrile (ACN)/methanol (MeOH) (7:3 = v:v) (Solvent A) and water (Solvent B) at a constant flow rate of 0.5 ml/min

	%A	%B	Flow rate (ml/min)
Initial	75	25	0.5
0.6	75	25	0.5
6.5	95.1	4.9	0.5
7.5	100	0	0.5
13.6	100	0	0.5
14.1	75	25	0.5
16.6	75	25	0.5

*Note*

UPLC: ultra‐performance liquid chromatographic.

### 2.5 DPPH radical scavenging assay

The ability of Cheongja#3 to scavenge free radicals was determined by the DPPH assay (Blois, 1958). Various concentrations of Cheongja#3 extract were dissolved in 40% EtOH and then mixed with equal volume of 0.2 mM DPPH solution (in EtOH). The mixtures were incubated at 37°C for 30 min. The absorbance was read at 517 nm (Spectramax M2e, Molecular devices, Sunnyvale, CA, USA). Results are expressed as electron donating ability (EDA) (%).

$$\text{EDA}\left(\%\right)=\frac{\text{Absorbance}\;\text{of}\;\text{control}-\text{Absorbance}\;\text{of}\;\text{samples}}{\text{Absorbance}\;\text{of}\;\text{control}}\times 100$$

### 2.6 Cell viability assay

Cell viability was measured by the MTT colorimetric assay (Mosmann, 1983). Briefly, cells were seeded in 96‐well plates and incubated overnight. Next, cells were pretreated with various concentrations of Cheongja#3. After 24 hr, cells were exposed to 500 μM of H_2_O_2_ for 24 hr. Five mg/ml of MTT solutions was added into the medium at a final concentration of 0.5 mg/ml, and incubated for 4 hr. All medium were removed and DMSO solution was added into each well to resuspend the MTT formazan. The absorbance was measured at 540 nm (Spectramax M2e, Molecular devices).

### 2.7 Measurement of intracellular ROS

Intracellular ROS were measured by the DCF‐DA fluorescence assay (LeBel, Ischiropoulos, & Bondy, 1992). Cells were grown in black well clear bottom 96‐well plates for 24 hr. Next, the cells were washed with phosphate‐buffered saline (PBS) two times and stained with 25 μM of DCF‐DA for 30 min. Subsequently, the cells were treated with soybean extracts in the absence or presence of H_2_O_2_ for 3 hr. The fluorescence was read at 485 (excitation)/535 (emission) nm (Spectramax M2e, Molecular devices).

### 2.8 Preparation of cytosolic and nuclear fraction

To separate cytosolic and nuclear fraction in cells, we followed previous study (Park et al., 2017). Cells were pretreated with Cheongja#3 extracted at concentrations of 10, 100 μg/ml for 24 hr. And then 500 μM of H_2_O_2_ was added to the cells for another 24 hr. Cells were collected and lysed with chilled lysis buffer A (20 mM Tris‐Cl at pH7.8, 1.5 mM MgCl₂, 10 mM KCl, 0.2 mM ethylenediaminetetraacetic acid [EDTA], 0.5 mM dithiothreitol [DTT], 6% NP‐40 and protease inhibitor cocktail). After centrifugation, the supernatant as cytosolic fraction was separated from pellet. Afterward, the pellets were lysed with buffer B (Buffer A containing 0.5 M DTT, 5% glycerol, 400 mM NaCl, protease inhibitor cocktail, and phosphatase inhibitor cocktail), for 30 min on ice. The samples were centrifuged at 18,000 g, 10 min, 4°C, and then supernatant as nuclear protein was transferred into fresh tubes for immunoblotting.

### 2.9 Immunoblotting

As previously reported (Lee, Han, et al., 2016), samples were lysed with a RIPA buffer containing 25 mM Tris‐HCl at pH7.6, 150 mM NaCl, 1% NP‐40, 1% sodium deoxycholate, 0.1% SDS and protease inhibitors. The supernatants were collected by centrifugation, and equal amounts of protein were mixed with 4× sample buffer (250 mM of Tris‐Cl at pH 6.8, 8% SDS, 40% glycerol, 8% β‐mercaptoethanol, and 0.01% bromophenol blue). Boiled samples were loaded into SDS‐PAGE gels and transferred onto PVDF membrane (Millipore, Billerica, MA, USA). The membranes were blocked with 5% skim milk for 1 hr at room temperature and reacted with primary antibodies overnight at 4°C. The PVDF membranes were then incubated with a horseradish peroxidase‐conjugated secondary antibody for 1 hr at room temperature. The protein band was developed using the enhanced chemiluminescence substrate.

### 2.10 Caspase‐3 activities assay (Colorimetric analysis)

Caspases‐3 activities were analyzed using colorimetric assay kit provided by Cayman chemicals, and all experiments were preformed according to the manufacturer's instruction. Briefly, cells were pretreated with Cheongja#3 for 24 hr and further incubated in H_2_O_2_ (500 μM) for 24 hr. After harvesting the cells, cells were lysed with lysis buffer provided in the kit. After centrifuge, supernatant was reacted with 1 M DTT and 4 mM DEVD‐p‐NA substrate for 2 hr at 37°C. The absorbance was read at 405 nm using microplate reader (Spectramax M2e, Molecular devices).

### 2.11 Statistical analysis

All experiments were performed in triplicate and expressed as mean ± *SD*. Data were analyzed using two‐tailed unpaired student's *t* test and considered as significant when *p* value under 0.05.

## 3 RESULTS

### 3.1 Fat‐soluble micronutrients contents in three soybean extracts

In Table 2, we determined tocopherols and carotenoid contents by UPLC analysis in three varieties of soybean extracts (Saedanbaek, Daechan, and Cheongja#3). γ‐Tocopherol was detected in Saedanbaek (2.6 mg/100 g) and Cheongja#3 (1.4 mg/100 g) ethanol extracts, but not in Daechan. In addition, Cheongja#3 extract had 9.9 μg/100 g of lutein while Saedanbaek had 1.2 μg/100 g of lutein.

**Table 2:** Fat‐soluble bioactive components in 40% EtOH extract of Saedanbaek, Daechan, and Cheongja#3. The 40% EtOH extract of soybeans were loaded on a C18 column. Tocopherols were detected at 292 nm and lutein detected at 450 nm

Cultivars	Soybean extracts		
	Saedanbaek	Daechan	Cheongja#3
γ‐Tocopherol (mg/100 g)	2.6	N.D.	1.4
Lutein (μg/100 g)	1.2	N.D.	9.9

*Note*

N.D.: Not detected.

### 3.2 Antioxidant activities of Cheongja#3

**Figure 1:**
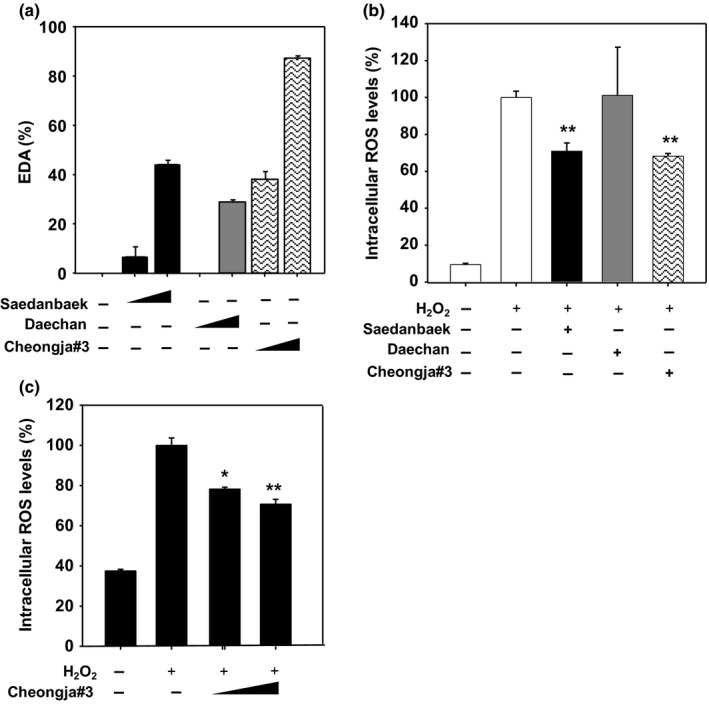
Antioxidant activities of soybean extracts. (a) In vitro DPPH free radical scavenging activities of Saedanbaek, Daechan, and Cheongja#3 extracts at concentrations of 1 and 10 mg/ml (b) Intracellular reactive oxygen species (ROS) levels assessed by DCF‐DA probe. Three varieties of soybean extracts at 100 μg/ml were treated in the presence of 100 μM of H_2_O_2_ for 3 hr in HaCaT cells. (c) Intracellular ROS levels assessed by DCF‐DA probe. Various concentrations (1, 10, 100 μg/ml) of Cheongja#3 extract were treated in the presence of 500 μM of H_2_O_2_ for 3 hr in HaCaT cells. Data are expressed as mean ± *SD*. *p < 0.05, **p < 0.01 versus H_2_O_2‐_treated cells. DPPH, 2,2‐Diphenyl‐1‐picrylhydrazyl

We have evaluated free radical scavenging activities using DPPH assay in these three varieties of soybean extracts. It was found that the Cheongja#3 soybean extract had the highest DPPH radical scavenging activity compared to the other soybean extracts (Figure 1a). Next, intracellular ROS levels have been determined in these three varieties of soybean extracts‐treated human keratinocytes HaCaT cells. The intracellular ROS levels were decreased to 70.84% and 68.21% by the treatment of the Saedanbaek and Cheongja#3 extracts, respectively. On the other hand, Daechan soybean extracts did not show any effect on the intracellular ROS levels (Figure 1b). Furthermore, the intracellular ROS levels were significantly decreased in Cheongja#3 extract‐treated HaCaT cells in a dose‐dependent manner (78.27% at 10 μg/ml and 70.7% at 100 μg/ml compared to vehicle‐treated cells) (Figure 1c). When treated with 1,000 μg/ml of Cheongja#3 extract, the ROS levels were similar to that of 100 μg/ml treatment in HaCaT cells (data not shown), suggesting enough concentration of antioxidant activities. From these results, we can conclude that the Cheongja#3 extract has strong antioxidant activities than the other varieties.

### 3.3 Protective effects of Cheongja#3 on oxidative stress‐induced apoptosis

**Figure 2:**
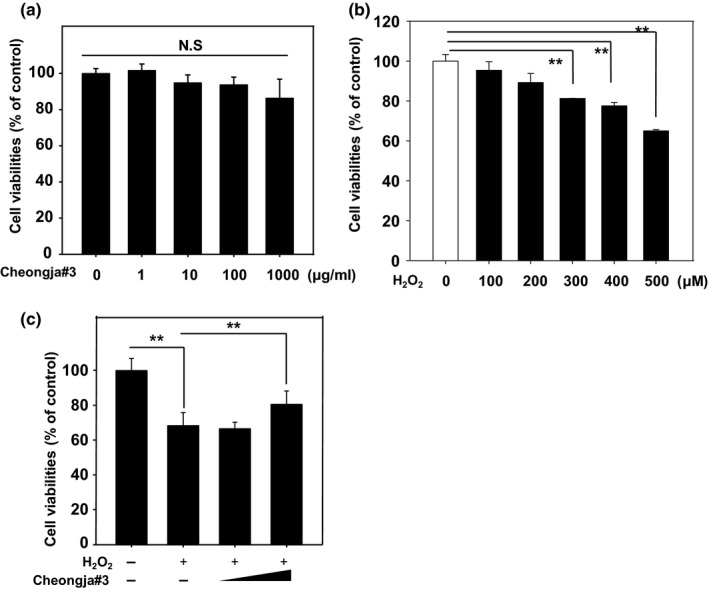
Effects of Cheongja#3 on cell viabilities. (a) Cells were treated with various concentrations (1, 10, 100, 1,000 μg/ml) of Cheongja#3 extracts for 24 hr followed by MTT assay to determine cell viabilities. (b) Cells were treated with various concentrations (0 to 500 μM) of hydrogen peroxide for 24 hr followed by MTT assay to determine cell viabilities. (c) Cells were pretreated with various concentrations (10, 100 μg/ml) of Cheongja#3 for 24 hr. Afterward medium containing extract was removed and further incubated in the presence of H_2_O_2_ for 24 hr followed by MTT assay to assess cell viabilities. Data are expressed as mean ± *SD*. **p < 0.01

**Figure 3:**
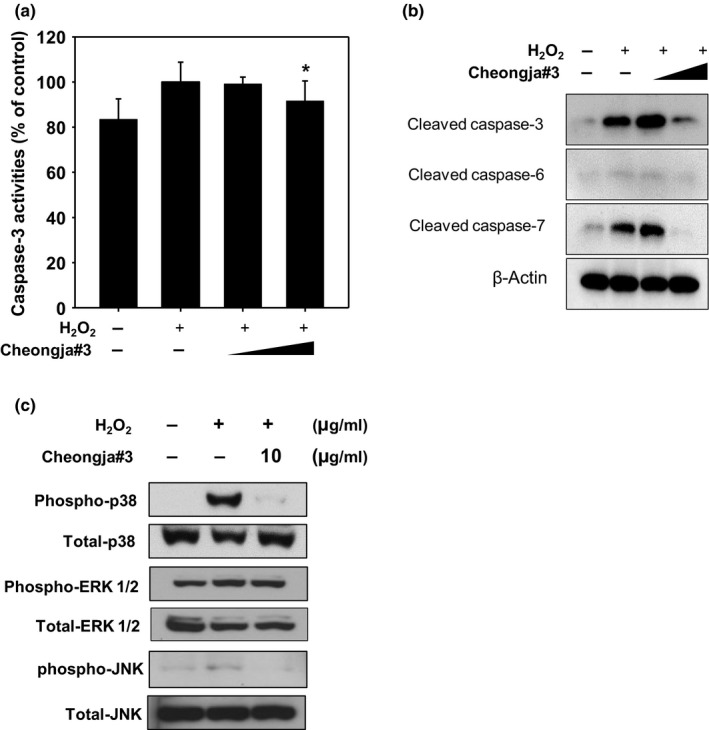
Effects of Cheongja#3 on caspases activities in HaCaT. (a) Cells were treated with Cheongja#3 (10, 100 μg/ml) for 24 hr. Afterward medium containing extract was removed and then treated with H_2_O_2_ for another 24 hr. Caspase‐3 enzymatic activities were determined in HaCaT cells. (b) Cleaved caspase‐3, caspase‐6, and, caspase‐7 protein levels were assessed by Western blotting. (c) MAPK protein p38, JNK, ERK protein levels were assessed by Western blotting. *p < 0.05 versus H_2_O_2‐_treated cells

Cheongja#3 extract had no cytotoxicity in the tested dose range (0–1,000 μM) for 24 hr (Figure 2a). And we found that exposure of cells to 500 μM of H_2_O_2_ resulted in sufficient cell death by approximately 65% as compared with vehicle‐treated cells (Figure 2b). In addition, pretreatment of Cheongja#3 soybean extract restored H_2_O_2_‐induced cell death in HaCaT cells (Figure 2c). To determine whether Cheongja#3 extract affected H_2_O_2_‐induced apoptotic cell death, we analyzed several apoptotic markers. As expected, H_2_O_2_‐treated cells show increased caspase‐3 enzymatic activities, whereas pretreatment of Cheongja#3 extract decreased H_2_O_2_‐activated caspases‐3 enzymatic activities (Figure 3a). Cleaved caspase‐3, caspase‐6, and, caspase‐7 protein levels were also decreased in Cheongja#3‐pretreated cells prior to exposure of H_2_O_2_ (Figure 3b). Since the MAPK pathway is the main signaling source for inducing apoptosis (Sui et al., 2014), we tested phosphorylated p38, phosphorylated extracellular‐signal‐regulated kinase (ERK) 1/2, phosphorylated c‐jun N‐terminal kinase (JNK) protein levels. Cheongja#3 extract downregulated H_2_O_2_‐induced phosphorylated p38 and JNK protein expression, whereas phosphorylated ERK 1/2 protein levels were not affected by Cheongja#3 extracts (Figure 3c).

### 3.4 Activation of Nrf‐2‐mediated HO‐1 by Cheongja#3

**Figure 4:**
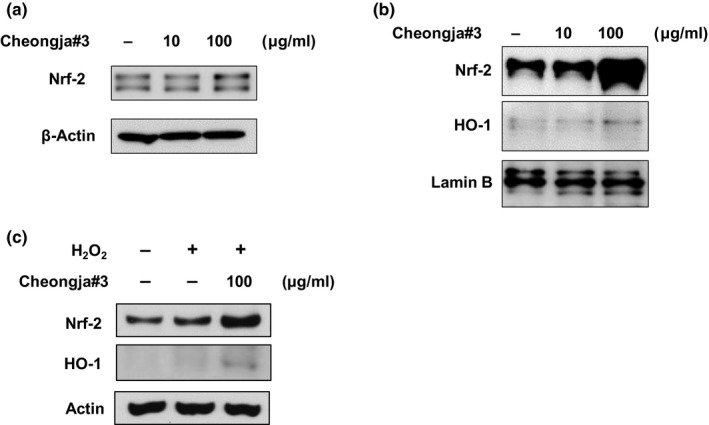
Effects of Cheongja#3 on antioxidant Nrf2 protein levels. (a) Cells were treated with 10, 100 μg/ml of Cheongja#3 for 24 hr. Nrf2 protein levels were assessed in total cell lysate using Western blotting (b). Nrf2 and HO‐1 protein levels were analyzed in the nucleus cell lysate using Western blotting (c) Cells were treated with Cheongja#3 extracts. Afterward medium containing extract was removed and then treated with H_2_O_2_ for another 24 hr. The Nrf2 and HO‐1 protein levels were determined by Western blotting

We further examined the mechanism of how Cheongja#3 extract attenuates H_2_O_2_‐induced apoptosis. Nrf‐2 protein expressions were increased in Cheongja#3‐treated cells in both total cell lysate (Figure 4a) and nucleus fraction of cells (Figure 4b). In addition, HO‐1, which is a gene downstream of Nrf2, was also increased by treatment of Cheongja#3 (Figure 4b). As expected, H_2_O_2_ slightly increased Nrf2 protein expression. We confirmed further increases of Nrf2 and HO‐1 protein expressions in Cheongja#3‐treated cells (Figure 4c).

## 4 DISCUSSION

In this study, we have demonstrated that black soybean, Cheongja#3, had effects on attenuating oxidative stress‐induced apoptosis via upregulating Nrf2‐mediated HO‐1 antioxidant system in human keratinocytes. Due to the abundant amounts of anthocyanins such as delphinidin‐3‐O‐glucoside, cyanidin‐3‐O‐glucoside, and petunidin‐3‐O‐glucoside in black soybean Cheongja#3, many studies have previously reported the health benefits of black soybeans such as antiadipogenic activities (Jeon et al., 2015; Kim, Kim, et al., 2012) and neuroprotective effects (Bhuiyan et al., 2012; Kim, Chung, et al., 2012). Additionally, γ‐tocopherol and lutein have been identified in Cheongja#3 extracts by UPLC. Tocopherols and carotenoids are well known for their antioxidant functions in prevention of various chronic diseases (Agarwal & Rao, 2000; Valentin & Qi, 2005). We believe that these fat‐soluble micronutrients in Cheongja#3 create a synergistic effect with the antioxidant functions of black soybeans in Cheongja#3 soybean extract than that is not found in other soybean varieties (Lee, Park, et al., 2015).

Many scientific literatures have reported that oxidative stress certainly can induce apoptosis in cells (Simon, Haj‐Yehia, & Levi‐Schaffer, 2000). Upon initiating apoptosis, cytochrome C is released from mitochondria and triggers cleavage of caspases (Bergmann, Yang, & Srivastava, 2003). We have detected cleaved caspase‐3, caspase‐6, and, caspase‐7 in the H_2_O_2_‐treated cells, but found antiapoptotic effects of Cheongja#3 by the rescued cleaved caspases. Mitogen‐activated protein kinase pathway, especially phosphorylated p38 and JNK, is en route to apoptosis in response to environmental stress such as ROS (Chen, Liu, Yin, Luo, & Huang, 2009; Sui et al., 2014). Phosphorylated JNK and phosphorylated p38 protein expressions were increased upon treatment of H_2_O_2_ as other studies have shown. On the other hand, Cheongja#3 extract restored upregulation of the protein expressions. Thus, the antiapoptotic effects of Cheongja#3 were regulated through phosphorylated p38 and JNK expression.

The transcription factor Nrf2 plays an important role in redox homeostasis via upregulation of its downstream antioxidant defense enzymes such as HO‐1. Nrf2 deficiency leads to various chronic diseases by failing detoxify environmental stresses such as medication, ingestion of food preserves, diesel exhaust, and others (Motohashi & Yamamoto, 2004). Since nuclear Nrf2 protein is a key contributor in antioxidant system, most of studies analyzed nuclear Nrf‐2 protein levels to assess antioxidant capacity. Our results have demonstrated increased Nrf‐2 and ‐mediated HO‐1 expression both in total cell lysate and nucleus fraction, denoting mechanism of antioxidant activity in Cheongja#3 extracts.

The epidermis, the most outer layer of body, undergoes frequent oxidative stress, resulting in a high incidence of skin diseases. In accordance with this, recent studies have reported that antioxidants such as echinacoside isolated from *Herba Cistanches* and liquiritin from Glycyrrhiza root reduce oxidative stress in keratinocytes—the major cell constituents of the epidermis in human and mice (Li et al., 2018; Zhang et al., 2017). Current study demonstrated that Cheongja#3, which is rich in antioxidants, and had a protective effect on oxidative stress, suggesting a promising functional food against skin diseases.

In summary, we demonstrated that Cheongja#3 extract had γ‐tocopherol and lutein in addition to anthocyanin, which is well‐studied previously. Cheongja#3 enhanced free radical scavenging activities and reduced intracellular ROS levels. It had protective effects on oxidative stress‐induced apoptosis by attenuating cleaved caspases and phosphorylated JNK and p38. Furthermore, they increased nucleus Nrf2 protein levels, thus proving its mechanism of antioxidant activities. Collectively, Cheongja#3 black soybean has biological function against oxidative stress in human keratinocytes.

## ETHICAL STATEMENT

This work does not involve any human or animal studies.

## CONFLICT OF INTEREST

None declared.
